# The optimal concentration of ropivacaine for transversus abdominis plane blocks in elective cesarean section: A protocol for systematic review and meta-analysis

**DOI:** 10.1371/journal.pone.0308335

**Published:** 2024-08-08

**Authors:** Xiangdong Zhang, Tangqi Qin, Donghang Zhang, Junwang Du

**Affiliations:** 1 Department of Anesthesiology, First People’s Hospital of Tianshui City, Tianshui, Gansu, China; 2 Department of Anesthesiology, The Second Affiliated Hospital, School of Medicine, The Chinese University of Hong Kong, Shenzhen & Longgang District People’s Hospital of Shenzhen, Shenzhen, Guangdong, China; 3 Department of Anesthesiology, West China Hospital, Sichuan University, Chengdu, Sichuan, China; The Warren Alpert Medical School of Brown University, Rhode Island Hospital, UNITED STATES OF AMERICA

## Abstract

**Introduction:**

Transversus abdominis plane (TAP) blocks are commonly performed for postoperative analgesia in elective cesarean section. Ropivacaine is the most commonly used local anesthetic for TAP blocks. Currently, the concentration of ropivacaine for TAP blocks is various, and increasing number of randomized controlled trials (RCTs) have compared the effects of different concentration of ropivacaine for TAP blocks in cesarean section. This protocol of a systematic review and meta-analysis aims to identify the optimal concentration of ropivacaine for TAP blocks in elective cesarean section.

**Methods and analysis:**

Databases including PubMed, Web of science, the Cochrane library, and EMBASE will be searched from their inception to May 1, 2024. RCTs that investigated the analgesia of different concentrations of ropivacaine for TAP blocks in elective cesarean section will be identified. The analgesia duration will be the primary outcome. Secondary outcomes will include the analgesics consumption over postoperative 24 hours, postoperative pain scores at rest and movement, and the incidence of adverse effects. RevMan 5.4 software will used for statistical analysis. The evidence quality of synthesized results will be evaluated by the Grading of Recommendations Assessment, Development, and Evaluation (GRADE) approach.

**Ethics and dissemination:**

Ethical approval is not applicable. The results of this study will be published on completion.

**Trial registration:**

PROSPERO registration number: CRD42024496907.

## Introduction

Postoperative pain is one of the most undesirable consequences for the patients after cesarean section [1]. Opioids are effective for pain management after cesarean section, but the related side effects, such as pruritus, constipation, sedation, dizziness, nausea and vomiting, tolerance, and respiratory depression limit its use [2,3]. Transversus abdominis plane (TAP) blocks can be easily performed and are commonly used for postoperative analgesia in cesarean section by reducing opioids consumption, improving pain scores, as well as decreasing the incidence of adverse effects [4–6]. Currently, ropivacaine is the most commonly used local anesthetic for TAP blocks due to less cardiovascular toxicity [7–9]. Various concentration of ropivacaine have been used for TAP blocks in cesarean section, including 0.25% [10,11], 0.35% [12], 0.375% [6,13], 0.5% [14], and 0.75% [15]. Previous evidence suggested that high concentration of ropivacaine for TAP blocks in cesarean section could increase the plasma concentrations of ropivacaine in patients, which might cause neurotoxicity [16]. Therefore, it is meaningful to determine the preferred concentration of ropivacaine which can provide longer analgesia without increasing the side effects in cesarean section. One meta-analysis revealed that 0.75% ropivacaine is the optimal choice for brachial plexus blocks in upper limb surgeries [17]. However, synthesized evidence from a systematic review and meta-analysis regarding the preferred concentration of ropivacaine for TAP blocks in cesarean section is not yet available. Therefore, we conducted this protocol of a systematic review and meta-analysis to identify the optimal concentration of ropivacaine for TAP blocks in cesarean section, which will provide evidence for the concentration selection of ropivacaine for TAP blocks in cesarean section.

## Methods and analysis

### Study registration

This protocol was previously registered in the International Prospective Register of Systematic Reviews (PROSPERO; ID: CRD42024496907) and was constructed according to guidelines of the Preferred Reporting Items for Systematic Evaluation and Meta-Analysis Protocols (PRISMA-P). The PRISMA-P-checklist is shown in S1 File.

### Search strategy

Databases including PubMed, Web of science, the Cochrane library, and EMBASE will be searched from their inception to May 1, 2024 with the language restriction to English. The key terms for search will contain “transversus abdominis plane”, “ropivacaine”, “cesarean section”, and “randomized controlled trials”. The detailed search plan for all databases is shown in S2 File. Additional studies will be identified via reviewing the citation lists of the relevant studies.

### Inclusion and exclusion criteria

Included RCTs should meet the following inclusion criteria: 1) Participants: patients underwent cesarean section, 2) Intervention and comparisons: analgesic effects between different concentrations of ropivacaine for TAP blocks, and 3) Primary outcomes: analgesia duration, which will be defined as the time interval from the completion of TAP block performance to the first request analgesia; secondary outcomes: the analgesics consumption over postoperative 24 hours, postoperative pain scores at rest and movement, and the incidence of adverse effects (e.g., nausea and vomiting, dizziness, drowsiness, itching, and constipation). Otherwise, studies will be excluded.

### Study selection

**Fig 1:**
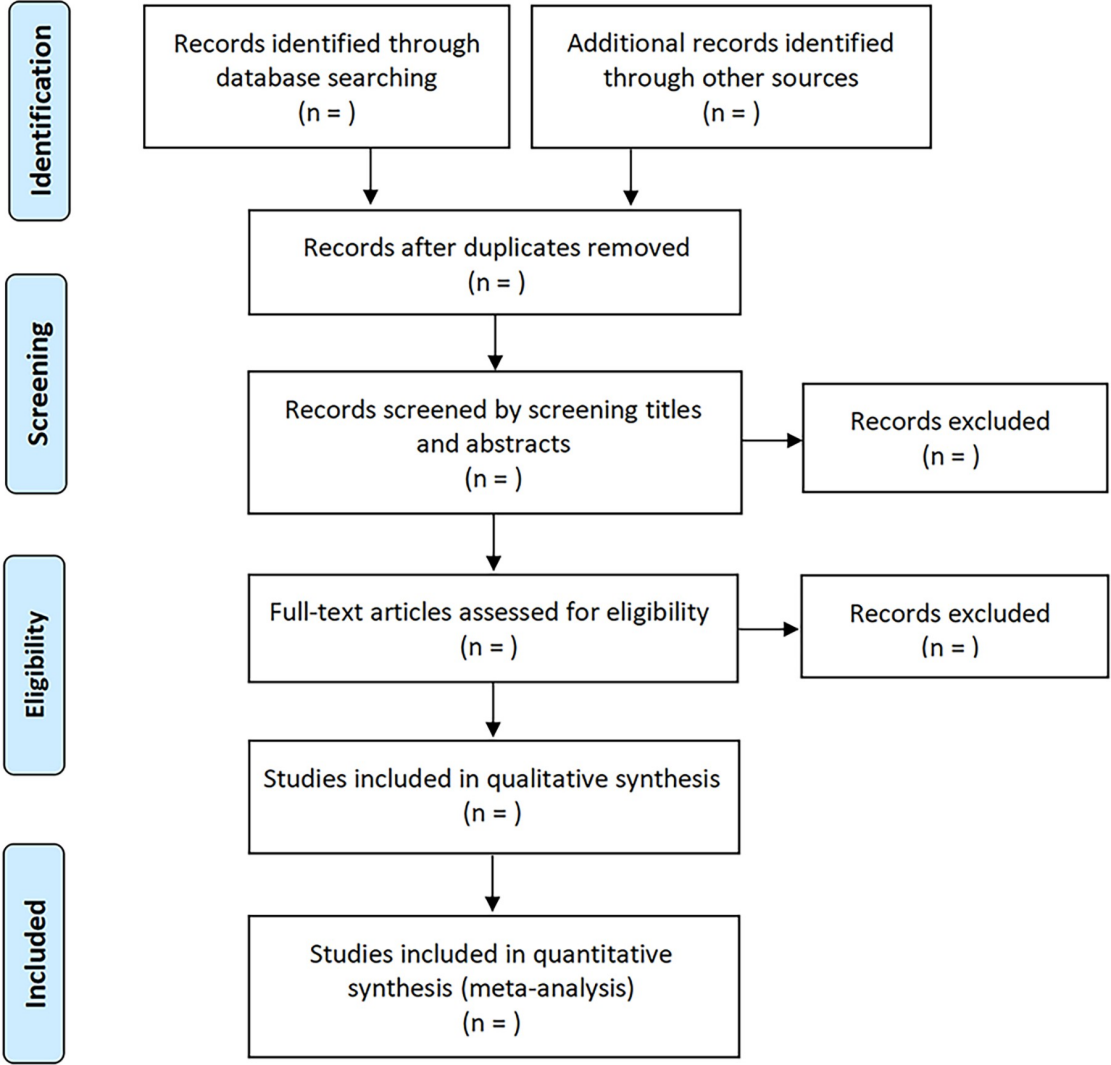
The flowchart of study selection.

The titles and abstracts of studies that identified by initial search will be independently screened by two authors (X.Z. and D.Z.). Then, the full text of potentially relevant studies will be reviewed to decide whether they conform to the inclusion criteria. Any discrepancy will be discussed with a third author (J.D.). The flowchart that will used for study selection was shown in Fig 1.

### Data extraction

Two authors (X.Z. and D.Z.) will independently extract the following information from included studies: authors, publication date, countries, characteristics of participants, sample size, anesthesia type, TAP approaches, adjuvants, surgical durations, interventions and controls, outcomes, and postoperative pain management. Any discrepancy will be discussed with a third author (J.D.).

### Risk of bias assessment

Two authors (X.Z. and D.Z.) will independently assess the risk of bias for each study using the the Cochrane Risk of Bias tool V.2 (RoB 2). Based on the random sequence generation, allocation concealment, blinding of participants and personnel, blinding of outcome assessment, incomplete outcome data, and selective reporting, the risk of bias will be rated as ‘some concerns’, ‘low’ or ‘high’. Any disagreement will be discussed with a third author (J.D.).

### Statistical analysis

Data processing and statistical analysis will be performed by RevMan 5.4. Continuous variables will be calculated using mean differences (MD) with 95% confidence intervals (CI), and dichotomous data will be presented using risk ratios (RR) with 95% CI. I^2^ test will be used to assess the statistical heterogeneity. A fixed-effect model will be used to pool data when I^2^ < 50%; otherwise, a random-effect model will be used and subgroup analysis and meta-regression will be applied to explore the heterogeneity source. Sensitivity analysis will also be used to test whether the pooled results are reliable. A *P* value less than 0.05 indicates statistical significance. Finally, the evidence quality will be assessed by the GRADE approach.

### Patient and Public Involvement

Not applicable.

### Ethics and dissemination

The ethical approval is not applicable. The results of this study will be publicly published on completion.

## Discussion

TAP blocks are commonly used for postoperative pain control in patients receiving elective cesarean section. Currently, various concentrations of ropivacaine are selected for TAP blocks in cesarean section. This protocol for a systematic review and meta-analysis aims to identify the optimal concentration of ropivacaine for TAP blocks in elective cesarean section, which will provide a longer postoperative analgesia without increasing the incidence of adverse effects.

Several concerns should be noticed. First, there might be a high clinical heterogeneity among included studies because of many factors, such as unilateral or bilateral TAP blocks, the approach and timing of TAP blocks, the adjuvants to ropivacaine, the surgical duration, the definition of outcomes, the anesthesia type and opioids use. Therefore, subgroup analysis or meta-regression should be used to explore the source of heterogeneity, and to determine the influence of different factors on the analgesic effects of different concentration of ropivacaine for TAP blocks in cesarean section. Another issue needs to be cautioned that the number of included studies and sample size might be small, which will influence the reliability of pooled results. Therefore, the GRADE approach will assess the quality of evidence for each outcome. Recently, laparoscopic TAP blocks were applied to provide postoperative analgesia in colorectal surgeries [18,19], it will be interesting to investigate the effects of laparoscopic TAP blocks in elective cesarean section. This protocol might serve as a reference method to investigate the optimal concentration of ropivacaine for other nerve blocks, such as quadratus lumborum block in cesarean section.

## Supporting information

S1 File The PRISMA-P-checklist. (DOC)

S2 File Search strategy for all databases. (DOCX)

## References

[1] Veef E, Van de Velde M: Post-cesarean section analgesia. Best Pract Res Clin Anaesthesiol 2022, 36(1):83–88. doi: 10.1016/j.bpa.2022.02.006 35659962

[2] Gadsden J, Hart S, Santos AC: Post-cesarean delivery analgesia. Anesth Analg 2005, 101(5 Suppl):S62–s69. doi: 10.1213/01.ANE.0000177100.08599.C8 16334493

[3] Tan HS, Habib AS: The optimum management of nausea and vomiting during and after cesarean delivery. Best Pract Res Clin Anaesthesiol 2020, 34(4):735–747. doi: 10.1016/j.bpa.2020.04.012 33288123

[4] Sultan P, Sultan E, Carvalho B: Regional anaesthesia for labour, operative vaginal delivery and caesarean delivery: a narrative review. Anaesthesia 2021, 76 Suppl 1:136–147. doi: 10.1111/anae.15233 33426655

[5] Staker JJ, Liu D, Church R, Carlson DJ, Panahkhahi M, Lim A et al: A triple-blind, placebo-controlled randomised trial of the ilioinguinal-transversus abdominis plane (I-TAP) nerve block for elective caesarean section. Anaesthesia 2018, 73(5):594–602. doi: 10.1111/anae.14222 29377066

[6] Jadon A, Jain P, Chakraborty S, Motaka M, Parida SS, Sinha N et al: Role of ultrasound guided transversus abdominis plane block as a component of multimodal analgesic regimen for lower segment caesarean section: a randomized double blind clinical study. BMC Anesthesiol 2018, 18(1):53. doi: 10.1186/s12871-018-0512-x 29759061 PMC5952861

[7] Plakhotnik J, Zhang L, Estrada M, Coles JG, Lonnqvist PA, Maynes JT: Local Anesthetic Cardiac Toxicity Is Mediated by Cardiomyocyte Calcium Dynamics. Anesthesiology 2022, 137(6):687–703. doi: 10.1097/ALN.0000000000004389 36170651

[8] Mather LE: The acute toxicity of local anesthetics. Expert Opin Drug Metab Toxicol 2010, 6(11):1313–1332. doi: 10.1517/17425255.2010.514265 20738226

[9] Hansen TG: Ropivacaine: a pharmacological review. Expert Rev Neurother 2004, 4(5):781–791. doi: 10.1586/14737175.4.5.781 15853505

[10] Yu Y, Gao S, Yuen VM, Choi SW, Xu X: The analgesic efficacy of ultrasound-guided transversus abdominis plane (TAP) block combined with oral multimodal analgesia in comparison with oral multimodal analgesia after caesarean delivery: a randomized controlled trial. BMC Anesthesiol 2021, 21(1):7. doi: 10.1186/s12871-020-01223-3 33413104 PMC7789306

[11] McKeen DM, George RB, Boyd JC, Allen VM, Pink A: Transversus abdominis plane block does not improve early or late pain outcomes after Cesarean delivery: a randomized controlled trial. Can J Anaesth 2014, 61(7):631–640. doi: 10.1007/s12630-014-0162-5 24764186

[12] Yan ZR, Chen LJ, Zhang SJ, Zhang LX, Lu H, Zhang L et al: The transversus abdominis plane block in conjunction with intrathecal morphine use after cesarean section in women with severe pre-eclampsia: a randomized controlled trial. BMC Anesthesiol 2023, 23(1):100. doi: 10.1186/s12871-023-02061-9 36997853 PMC10061731

[13] Dereu D, Savoldelli GL, Mercier Y, Combescure C, Mathivon S, Rehberg B: The impact of a transversus abdominis plane block including clonidine vs. intrathecal morphine on nausea and vomiting after caesarean section: A randomised controlled trial. Eur J Anaesthesiol 2019, 36(8):575–582. doi: 10.1097/EJA.0000000000001013 31274545

[14] Lee AJ, Palte HD, Chehade JM, Arheart KL, Ranasinghe JS, Penning DH: Ultrasound-guided bilateral transversus abdominis plane blocks in conjunction with intrathecal morphine for postcesarean analgesia. J Clin Anesth 2013, 25(6):475–482. doi: 10.1016/j.jclinane.2013.05.004 24012493

[15] Kanta B, Sonali D, Gazala P, Yunus K, Kiran K: A randomised comparative study of transversus abdominis plane block with or without intravenous diclofenac sodium as a component of multimodal regimen for post-operative analgesia following caesarean section. Indian J Anaesth 2021, 65(4):316–320. doi: 10.4103/ija.IJA_761_20 34103746 PMC8174596

[16] Griffiths JD, Le NV, Grant S, Bjorksten A, Hebbard P, Royse C: Symptomatic local anaesthetic toxicity and plasma ropivacaine concentrations after transversus abdominis plane block for Caesarean section. Br J Anaesth 2013, 110(6):996–1000. doi: 10.1093/bja/aet015 23454825

[17] Wu L, Zhang W, Zhang X, Wu Y, Qu H, Zhang D et al: Optimal concentration of ropivacaine for brachial plexus blocks in adult patients undergoing upper limb surgeries: a systematic review and meta-analysis. Front Pharmacol 2023, 14:1288697. doi: 10.3389/fphar.2023.1288697 38035018 PMC10687368

[18] La Regina D, Popeskou SG, Saporito A, Gaffuri P, Tasciotti E, Dossi R et al: Laparoscopic versus ultrasound-guided transversus abdominis plane block in colorectal surgery: a non-inferiority, multicentric randomized double-blinded clinical trial. Colorectal Dis 2023, 25(9):1921–1928. doi: 10.1111/codi.16689 37525414

[19] Zaghiyan KN, Mendelson BJ, Eng MR, Ovsepyan G, Mirocha JM, Fleshner P: Randomized Clinical Trial Comparing Laparoscopic Versus Ultrasound-Guided Transversus Abdominis Plane Block in Minimally Invasive Colorectal Surgery. Dis Colon Rectum 2019, 62(2):203–210. doi: 10.1097/DCR.0000000000001292 30540660

