# Supplementary material for: The optimal concentration of ropivacaine for transversus abdominis plane blocks in elective cesarean section: A protocol for systematic review and meta-analysis
Source: PLoS One. 2024 Aug 8;19(8):e0308335. doi: 10.1371/journal.pone.0308335 (PMC11309379; doi:10.1371/journal.pone.0308335)
Supplement: S2 File — (DOCX) [file pone.0308335.s002.docx]

**Search strategies**

**PubMed**

#1 transversus abdominis plane block [Title/Abstract]

#2 TAP block [Title/Abstract]

#3 #1 OR #2

#4 cesarean section [Mesh]

#5 cesarean section [Title/Abstract]

#6 caesarean section [Mesh]

#7 caesarean section [Title/Abstract]

#8 cesarean delivery [Mesh]

#9 cesarean delivery [Title/Abstract]

#10 caesarean delivery [Mesh]

#11 caesarean delivery [Title/Abstract]

#12 #4 OR #5 OR #6 OR #7 OR #8 OR #9 OR #10 OR #11

#13 ropivacaine [Mesh]

#14 ropivacaine [Title/Abstract]

#15 #13 OR #14

#16 randomized controlled trial [Title/Abstract]

#17 randomized controlled study [Title/Abstract]

#18 controlled clinical trial [Title/Abstract]

#19 clinical trial [Title/Abstract]

#20 clinical study [Title/Abstract]

#21 randomized [Title/Abstract]

#22 #16 OR #17 OR #18 OR #19 OR #20 OR #21

#23 #3 AND #12 AND #15 AND #22

**EMBASE**

#1 ‘transversus abdominis plane block’/exp OR ‘transversus abdominis plane

block’:ti,ab,kw OR ‘TAP block’:ti,ab,kw

#2 ‘cesarean section’/exp OR ‘cesarean section’:ti,ab,kw OR ‘caesarean section’:ti,ab,kw OR ‘cesarean delivery’:ti,ab,kw OR ‘caesarean delivery’:ti,ab,kw

#3 ‘ropivacaine’/exp OR ‘ropivacaine’:ti,ab,kw

#4 ‘randomized controlled trial’/exp OR ‘randomized controlled trial’:ti,ab,kw OR

‘randomized controlled study’:ti,ab,kw OR ‘controlled clinical trial’:ti,ab,kw OR

‘clinical trial’:ti,ab,kw OR ‘clinical study’:ti,ab,kw OR ‘randomized’:ti,ab,kw

#5 #1 AND #2 AND #3 AND #4

**Cochrane Library**

#1 (transversus abdominis plane block):ti,ab,kw

#2 (TAP block):ti,ab,kw

#3 #1 OR#2

#4 MeSH descriptor: (cesarean section) explode all trees

#5 (cesarean section):ti,ab,kw

#6 (caesarean section):ti,ab,kw

#7 (cesarean delivery):ti,ab,kw

#8 (caesarean delivery):ti,ab,kw

#9 #4 OR #5 OR #6 OR #7 OR #8

#10 MeSH descriptor: (ropivacaine) explode all trees

#11 (ropivacaine):ti,ab,kw

#12 #10 OR #11

#13 (randomized controlled trial):ti,ab,kw

#14 (randomized controlled study):ti,ab,kw

#15 (controlled clinical trial):ti,ab,kw

#16 (clinical trial):ti,ab,kw

#17 (clinical study):ti,ab,kw

#18 (randomized):ti,ab,kw

#19 #13 OR #14 OR #15 OR #16 OR #17 OR #18

#20 #3 AND #9 AND #12 AND #19

**Web of Science**

#1 transversus abdominis plane block (Topic) OR TAP block (Topic)

#2 cesarean section (Topic) OR caesarean section (Topic) OR cesarean delivery (Topic) OR caesarean delivery (Topic)

#3 ropivacaine (Topic)

#4 randomized controlled trial (Topic) OR randomized controlled study (Topic) OR

controlled clinical trial (Topic) OR clinical trial (Topic) OR clinical study (Topic) OR

randomized (Topic)

#5 #1 AND #2 AND #3 AND #4
